# Microbial Colonization of the Host Plant: Cellular and Molecular Mechanisms of Symbiosis

**DOI:** 10.3390/ijms25010639

**Published:** 2024-01-04

**Authors:** Elena E. Fedorova, José J. Pueyo

**Affiliations:** 1 Timiryazev Institute of Plant Physiology, Russian Academy of Science, 127276 Moscow, Russia; 2 Department of Soil, Plant and Environmental Quality, Institute of Agricultural Sciences, ICA-CSIC, 28006 Madrid, Spain; jj.pueyo@csic.es

Nitrogen is an essential element for all plants, animals, and microorganisms in the Earth’s biosphere. For millions of years, nitrogen was available through soil minerals; dead organisms; and biological nitrogen fixation by microorganisms, with symbiotic nitrogen fixation in particular being the most efficient source. Humans have used legumes and symbiotic nitrogen fixation in all continents for thousands of years without knowing anything about symbiosis. Back in Roman times, in the literature on soil improvement, the positive impact on yields and soil quality after planting legumes was already noted. The 20th century industrial technology of chemical synthesis increased the availability of nitrogenous fertilizers. Thanks to this, crop yields improved significantly, and the human mortality rate from starvation reduced. Nitrogen fertilizers are expensive and not quite ecologically friendly, but, considering the size and growth of the human population, they are strictly necessary. A trend in technology of recent times has been the search for environmentally friendly methods to replace the “dirty” technologies used since the beginning of the technological era. This has led to the development of research in biological nitrogen fixation [1]. Over the past 30–40 years, studies on legume–rhizobium symbiosis have obtained a vast amount of information regarding the structure of root nodules and the dynamics of symbiosis formation. The scientific community working in this area of research was fascinated by the logic of the successive steps in the establishment of symbiosis and by the cytological beauty of root nodules, as it was almost like Newton’s celestial mechanics. The advent of new methods of genetics and molecular biology revealed complexity in seemingly simple processes such as inoculation, plant selection of microsymbionts, and nodule formation. In return, researchers gained the ability to manipulate both plant and bacterial genes instead of being passive spectators [1,2]. Major efforts have been made to decipher Nod factor (NF) signaling pathways and the mechanisms of the perception of NF in susceptible regions of young roots, as well as the regulation of nodule formation [2,3,4].

The idea underlying these efforts was appealing. It was hypothesized that the transfer of a megaplasmid containing the genes responsible for the Nod factor signaling pathway into a non-legume plant would be a sufficient step to create a new symbiosis. Todate, this approach has not yet achieved the expected results, but the hope for a bright future is still alive. Genomics research on the non-legume tree *Parasponia*, which represents a relatively young symbiosis in evolution terms, has helped to formulate a new hypothesis concerning the evolution of symbiotic relationships. It was proposed that nodulation evolved only once in an ancestor of the nitrogen-fixing clade and that this trait was subsequently lost in most descendant lineages, probably due to a decrease in the carbon dioxide content in the atmosphere, which made nitrogen fixation unprofitable for the host plant. Velzen et al. [5] showed that the genetic control of the process of nodule organogenesis and the geneticcontrol of the process of colonization of the host plant tissue were carried out independently. The study of nodule formation expanded to genomic and transcriptomic analyses, revealing the associated transcriptional reprogramming [6]. Root nodule proteomics and metabolomics uncovered the biological meaning of root nodule zonation, which was established from root nodule anatomy through cytological analyses. It was shown that the shift from sugars to dicarboxylic acids as the main carbon source for nitrogen fixation by bacteroids starts in the infection zone and further expands to the nitrogen-fixing zone [7]. A large number of transcription factors have been implicated in one or more stages of symbiosis and in the response to environmental changes affecting symbiosis, including biotic and abiotic stresses [8,9,10].

Our Special Issue on “Microbial colonization of the host plant: cellular and molecular mechanisms of symbiosis” has attracted the attention of several research groups, and we are very grateful for their participation. These groups work in different research areas of symbiosis, and their valuable contributions have made this Special Issue broader and more informative, reflecting the up-to-date developments in the field. Two excellent reviews were presented by Jiménez-Guerrero and co-workers [11] and by Grundy and co-workers [12], describing some of the defense and protective mechanisms in symbiosis. The work of Jiménez-Guerrero et al. [11] centered on the role of the type 3 secretion system (T3SS) and its importance in certain rhizobium–legume symbioses. The authors provided an analysis of the literature concerning the molecular signals during the development of symbiosis, when rhizobia synthetize molecular signals that are recognized by the plant to ensure the progression of symbiosis by suppressing and overcoming the plant immune responses. In summary, the authors emphasize that rhizobial type 3, 4, and 6 secretion systems might have a positive, neutral, or negative effect on symbiosis. The main function of these secretion systems is to suppress plant defense responses to facilitate bacterial infection. They described in detail the role of T3SS in soybean; in several *Vigna* and *Lotus* species; and in the rather unusual symbiosis of *Aeschynomene* species nodulated by *Bradyrhizobium* strains, which possess a functional T3SS but are incapable of producing NF. Thus, the authors were able to compare the symbiotic phenotypes of T3SS published todate and classify them into neutral, positive, or negative, which allowed them to name T3SS as the Dr. Jekyll and Mr. Hyde of symbiosis.

The review by Grundy et al. [12] centered on a very important, yet not sufficiently studied, topic of the role of the plant immune system in nodulation. The review focused on the cycles of microbe-associated molecular-pattern-triggered immunity and effector-triggered immunity. The authors provided a comprehensive analysis of the literature concerning the immune reaction during symbiosis development, the transient and local defense responses induced in the host plant, and the mechanisms rapidly suppressing the defense responses. They highlighted the alternatives in such reactions, a lack of defense reactions in the case of inoculation with compatible rhizobia in *Lotus* and *Aeschynomene* symbioses and the defense signaling induced by rhizobia and NF in *Medicago sativa*. *Sinorhizobium meliloti* inoculation triggers the production of active oxygen species, similar to what occurs in the response to pathogen presence. They summarized the data concerning microbe-associated molecular patterns (MAMPs) and the inductors of such reactions, as well as the evasion of MAMP-triggered immunity, providing a list of legume proteins with functions in defense reactions. They also described the role of NF in the suppression of defense reactions in different plants, with an explanation of the putative mechanisms involved. Grundy et al. [12] also described the role of thetype 3 secretion system and NF-dependent and independent effectors. Quite interesting is the sub-section concerning the receptors termed R proteins and their crucial role in determining hostspecificity by limiting the number of strains that can successfully infect the host. The authors also discussed the information concerning the role of effector perception and immune signaling in the calcium signaling that regulates legume–rhizobium symbiosis, emphasizing the necessity for further investigations on the plant immune system’s role in rhizobial infection mechanisms.

Another remarkable review presented by Msaddak et al. [13] was dedicated to the data concerning the symbiosis of *Lupinus* species. Lupin is a high-protein legume crop that grows under a wide range of edaphoclimatic conditions where other crops are not viable. Its unique seed nutrient profile can promote health benefits, and it has been proposed as a phytoremediation plant. Based on the available data, the authors related that most rhizobia-nodulating *Lupinus* species belong to the genus *Bradyrhizobium*, comprising strains that are phylogenetically related to *B. cytisi*, *B. hipponenese*, *B. rifense*, *B. iriomotense*/*B. stylosanthis*, *B. diazoefficiens*, *B. japonicum*, *B. canariense*/*B. lupini*, and *B. retamae*/*B. valentinum*. However, lupins are also nodulated by fast-growing bacteria within the genera *Microvirga*, *Ochrobactrum*, *Devosia, Phyllobacterium*, *Agrobacterium*, *Rhizobium*, and *Neorhizobium*. Phylogenetic analyses of the *nod* and *nif* genes, involved in microbial colonization and symbiotic nitrogen fixation, respectively, suggested that these fast-growing lupin-nodulating bacteria acquired their symbiotic genes from rhizobial genera other than *Bradyrhizobium*. Horizontal transfer represents a key mechanism that allows lupin to form symbioses with bacteria that were previously considered as non-symbiotic or unable to nodulate this species, a fact that might favor lupins’ adaptation to specific habitats. The characterization of yet-unstudied *Lupinus* species, including microsymbiont whole-genome analyses, will most likely expand and modify the current lupin microsymbiont taxonomy and provide additional knowledge that might help in further increasing lupins’ adaptability to marginal soils and climates.

Brito-Santana and co-workers [14] presented experimental data describing the role of the *dnaJ* gene of *Sinorhizobium meliloti* in efficient nodulation. The paper centered on the very early steps of symbiosis and the directed movement of bacteria toward the roots, followed by the attachment of bacteria to the root surface. The authors used the interesting flagella-minus and flagella-plus *dnaJ* deletion mutants, revealing the role of this gene in surface translocation rather than swimming. The DnaJ protein belongs to the ATP-dependent chaperone folding system DnaK/DnaJ/GrpEand, and it functions as a chaperone and holdase that transfers unfolded, misfolded, or aggregated proteins to DnaK, which is responsible for folding with the participation of the nucleotide exchange factor GrpE. The role of *S. meliloti* DnaJ was analyzed in response to oxidative stress, saltstress, and the establishment of symbiosis with alfalfa. The results obtained in this work demonstrate that *S. meliloti* DnaJ plays an important role in the establishment of efficient symbiosis with alfalfa plants. The data showed that this chaperone is required at early and late stages of symbiosis, such as nodule formation and nodule colonization. The symbiotic performance exhibited by three *dnaJ* mutants correlated with their tolerance to stress. Besides DnaJ, additional chaperones have been shown to be crucial for effective symbiosis with legumes.

Trifonova and co-workers [15] described the effects caused by intracellular nitrogen-fixing bacteria in host cells. As it turns out, conditions in the root nodule are not completely optimal, and the host cells lack potassium due to the inhibition of activity and the cessation of the functioning of potassium transporters. Confocal and electron microscopy immunolocalization showed that proteins related to potassium transport and Na^+^/K^+^ exchangers in the root nodule, including the plasma membrane exchanger MtNHX7 and the endosome/tonoplast exchanger MtNHX6, were depleted from their target membranes and expelled to the vacuoles in infected cells. This mistargeting suggested the partial loss of the exchangers’ functionality. In the mature part of the nodule, 7 of the 20 genes encoding ion transporters, channels, and Na^+^/K^+^ exchangers were either not expressed or substantially downregulated, putatively due to the partial hypoxic conditions in the infected zone of the nodule. In nodules from plants subjected to salt treatments, low-temperature scanning electron microscopy and X-ray microanalysis revealed the accumulation of 5–6 times more Na+ in infected cells than in non-infected cells. Hence, the infected cells’ inability to withstand salt stress may be the integral result of preexisting defects in the localization of proteins involved in Na^+^ exclusion and the reduced expression of key genes involved in ion homeostasis, resulting in premature senescence and the termination of symbiosis.

Finally, Fedorova’s review [16] focused on the changes that occur in the endomembrane system of infected cells and the putative mechanisms involved in infected cells’ adaptation to their unusual lifestyle. The changes in the endomembranes of infected cells occur at each stage of symbiosis, from the architecture of the root hair after contact with rhizobia to the formation of lytic compartments in the zone of symbiosis termination. The special ecological niche for living nitrogen-fixing bacteria in root nodules is based on the specific membrane interface. Due to the presence of endocytotic markers on the membrane, symbiosomes are able to survive and fix atmospheric nitrogen for several days, the time required to fulfill their task inside the infected cell. The conditions in the infected cells’ environment, such as partial hypoxia, rapid changes in membrane and cytoskeleton configuration, a lack of sugars, changes in vacuole functionality, and the redirection of vesicle traffic, as well as the drastic changes in gene expression, affect the endomembrane system of infected cells, and the time of retention and the functionality of transport proteins in the membranes. The features of an infected cell’s endomembrane system must be taken into consideration in works aiming to improve certain aspects of the symbiotic relationship via the creation of genetically modified mutants with altered expression levels of certain genes.

During the last 20 years, the study of symbiotic relationships in plants has made great progress. We can point to the tremendous advances in research on signaling between partners in the early stages of symbiosis and in transcriptomics research, which has made it possible to understand the changes in gene expression during symbiosis. However, a great number of processes that are related to the regulation of membrane transport, protein targeting, changes in the cytoskeleton, and the regulation of osmotic processes in infected cells are still waiting to be explored. New tools, such as genomic, transcriptomic, microbiome, and secretome analyses, have revealed the complexity of the mutual influence between symbiotic partners, as well as the significant role of environmental conditions in the interaction of symbiotic partners. Working on symbiosis, we are aware of the limitations and the disadvantages of its practical application. These include the negative effect of the intracellular colony of rhizobia on the host cell’s welfare, the need for highly effective plant- and environment-adapted strains of rhizobia, and the need for varieties of host plants highly efficient in symbiosis. We know that symbiosis efficiency is restricted by environmental conditions. We also know that the supply of nitrogen provided by symbiosis is limited by the autoregulation of nodule formation and that the host plant is provided with the physiological minimum amount of nitrogen. This minimum may keep the host plant alive, but it may not be sufficient to support high-yielding legume crops. Most importantly, we know that symbiosis is a short-term phenomenon, and its duration might not be enough to maintain a plant during its whole lifespan until seed maturation is completed.

Whatever the adversities, we also know that only the one who walks will master the road.
